# Towards a TDP-43-Based Biomarker for ALS and FTLD

**DOI:** 10.1007/s12035-018-0947-6

**Published:** 2018-02-19

**Authors:** Emily Feneberg, Elizabeth Gray, Olaf Ansorge, Kevin Talbot, Martin R. Turner

**Affiliations:** 1 Nuffield Department of Clinical Neurosciences, University of Oxford, Oxford, UK; 2 John Radcliffe Hospital, West Wing Level 6, Oxford, OX3 9DU UK; 3 Neuropathology, Nuffield Department of Clinical Neurosciences, University of Oxford, Oxford, UK

**Keywords:** TDP-43, Biomarker, Cerebrospinal fluid, Amyotrophic lateral sclerosis, Frontotemporal dementia, TARDBP

## Abstract

TDP-43 accumulates in nerve cells of nearly all cases of amyotrophic lateral sclerosis (ALS; the commonest form of motor neuron disease) and in the majority of Tau-negative frontotemporal lobar degeneration (FTLD). There is currently no biochemical test or marker of disease activity for ALS or FTLD, and the clinical diagnosis depends on the opinion of an experienced neurologist. TDP-43 has a key role in the pathogenesis of ALS/FTLD. Measuring TDP-43 in easily accessible biofluids, such as blood or cerebrospinal fluid, might reduce diagnostic delay and offer a readout for use in future drug trials. However, attempts at measuring disease-specific forms of TDP-43 in peripheral biofluids of ALS and FTLD patients have not yielded consistent results, and only some of the pathological biochemical features of TDP-43 found in human brain tissue have been detected in clinical biofluids to date. Reflecting on the molecular pathology of TDP-43, this review provides a critical overview on biofluid studies and future directions to develop a TDP-43-based clinical biomarker for ALS and FTLD.

## Introduction

TDP-43 has been identified as binding to the transactivation-responsive region of the HIV-1 gene, acting as transcriptional regulator [1]. It is a ubiquitous protein whose specific functions are probably crucial to establishing its pathogenic role linked to neurodegenerative diseases [2]. Amyotrophic lateral sclerosis (ALS; the commonest phenotype of motor neuron disease) is a neurodegenerative disease with a median survival from symptom onset of 30 months [3]. ALS is known to have clinical, pathological, and genetic overlap with frontotemporal dementia (FTD) [4]. Cytoplasmic neuronal and glial inclusions of TDP-43 are found in 98% of all cases of ALS (sporadic and familial) and in the majority of Tau-negative frontotemporal lobar degeneration (FTLD-TDP) cases, typically behavioral variant frontotemporal dementia (FTD) [5]. The diagnosis of ALS is currently made clinically in tertiary referral centers with a mean diagnostic delay from symptom onset of more than 1 year, when disease is well established and the potential therapeutic window may have passed. Therapeutic trials in ALS currently rely on survival or rate of change of disability scores as outcome measures which are insensitive and result in lengthy studies. A more objective and rapid diagnostic test would facilitate access to therapeutic trials for those suspected to have the disease but with more limited clinical signs. Neurochemical markers would ideally also predict disease progression and assess response to treatment. Such a biomarker might provide evidence of drug target engagement if it is proximal to the pathological process, and enable pre-symptomatic screening, to take advantage of the optimal therapeutic window for disease-modifying therapies. This review will provide an overview of progress towards the development of a TDP-43-based biomarker reflecting the most consistent molecular finding in ALS and FTLD.

## The Pathological Role of TDP-43 in ALS and FTLD

**Fig. 1:**
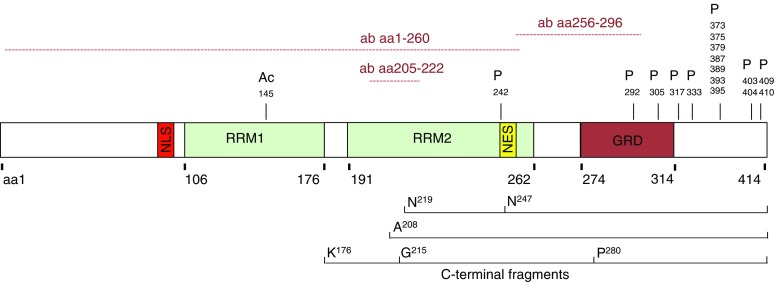
Schematic of full-length TDP-43 illustrating the important domains and C-terminal fragments found in ALS/FTD. *NLS* nuclear localization signal, *RRM* RNA-recognition motifs, *NES* nuclear export signal, *GRD* glycine-rich domain, *P* phosphorylation sites [8, 9], *Ac* acetylation site [10] and C-terminal fragments from Arg^208^ extracted from a 22-kDa gel band of sarkosyl-insoluble, urea-soluble FTLD-TDP brains [11], Asp (N)^219^ and Asp^247^ extracted from a 23-kDa band of FTLD-TDP [12] and from Lys (K)^176^, Gly^215^, Pro^280^ extracted from 23- to 25-kDa bands of ALS brain fractions [8]. *Aa* amino acid and *ab* antibody binding site

Cytoplasmic aggregation of ubiquitinated, phosphorylated, and truncated TDP-43 is a unifying pathologic observation across the clinical spectrum of ALS and FTLD [6, 7]. TDP-43 is a heterogeneous nuclear ribonucleoprotein (hnRNP) with a length of 414 amino acids (Fig. 1). It has various domains including two highly conserved RNA-recognition motifs (RRM) and a C-terminal glycine-rich domain with a high glutamine/asparagine content [13, 14]. The C-terminal region of TDP-43 is important for binding to homotypic and heterotypic proteins. It regulates important cellular functions such as splicing activity through formation of a complex with other hnRNPs and has a self-regulatory transcriptional function [15, 16]. The C-terminal region of TDP-43 is also classified as a “low complexity domain,” which is important in mediating liquid–liquid phase transitions in the protein important for the biogenesis of various membraneless organelles such as stress granules, but also renders it prone to misfolding and aggregation [17]. TDP-43 can be N-terminal truncated into C-terminal fragments that are often found in cytoplasmic aggregates along with the full-length form of TDP-43 [6]. The generation of shorter fragments may involve proteolytic cleavage, as has been described for TDP-43 through a caspase- or calcium-mediated calpain-protease [18] or alternate splicing [19].

**Fig. 2:**
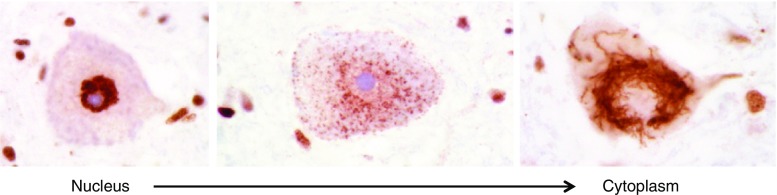
TDP-43 mislocalization from the nucleus into the cytoplasma and aggregate formation in motor neurons of the spinal cord of amyotrophic lateral sclerosis

Under physiological conditions, TDP-43 is a mainly nuclear protein. In most cases of ALS and FTLD, it is found translocated to the cytoplasm where it changes formation and forms aggregates (Fig. 2). Whether this occurs through a disruption of nuclear import or conformational change in the protein is not yet clear [20–23].

Support for a direct mechanistic link between TDP-43 and neurodegeneration came from the identification of mutations in the TAR-DNA binding protein (*TARDBP*) gene in hereditary forms of ALS, clinically and pathologically identical to sporadic cases [24–32]. However, only rare cases of *TARDBP* mutations linked to FTD without ALS have been described [33, 34]. Most ALS-causing genotypes are dominant missense mutations located in the C-terminal region of TDP-43, though one truncating mutation has been described [29]. These findings suggest a fundamental pathological function for misprocessing of TDP-43 in ALS and FTLD [35, 36].

Whether a loss of function of TDP-43 with impaired RNA-binding capacity and splicing dysfunction, or its mislocalization and aggregation resulting in a toxic gain of function, or both, cause ALS is still a matter of debate [37, 38]. Over-expression models of human mutant or C-terminal fragments of TDP-43 show disease-specific changes including nuclear clearing of endogenous TDP-43, cytoplasmic mislocalization, phosphorylation, and ubiquitination of aggregated TDP-43 accompanied by neuronal death [11, 39, 40]. Induced mislocalization of TDP-43 through over-expressing wild type or mutant TDP-43 results in cytoplasmic toxicity in the absence of the formation of inclusion bodies or extensive nuclear clearance of TDP-43 [41–43]. Complete knockout of TDP-43 is cell-lethal, but partial knockdown seems to directly impair endosomal pathways, which are necessary to regulate dendrite growth and neuronal signaling [44]. The role of full-length TDP-43 or its truncated C-terminal fragments in cellular toxicity is still debated as both have a propensity to form cytosolic aggregates [11, 45], but reduction of calpain-dependent cleavage of phosphorylated TDP-43 has an adverse effect on the degradation of TDP-43 [46].

## Neuropathology of TDP-43-Related Proteinopathies

**Fig. 3:**
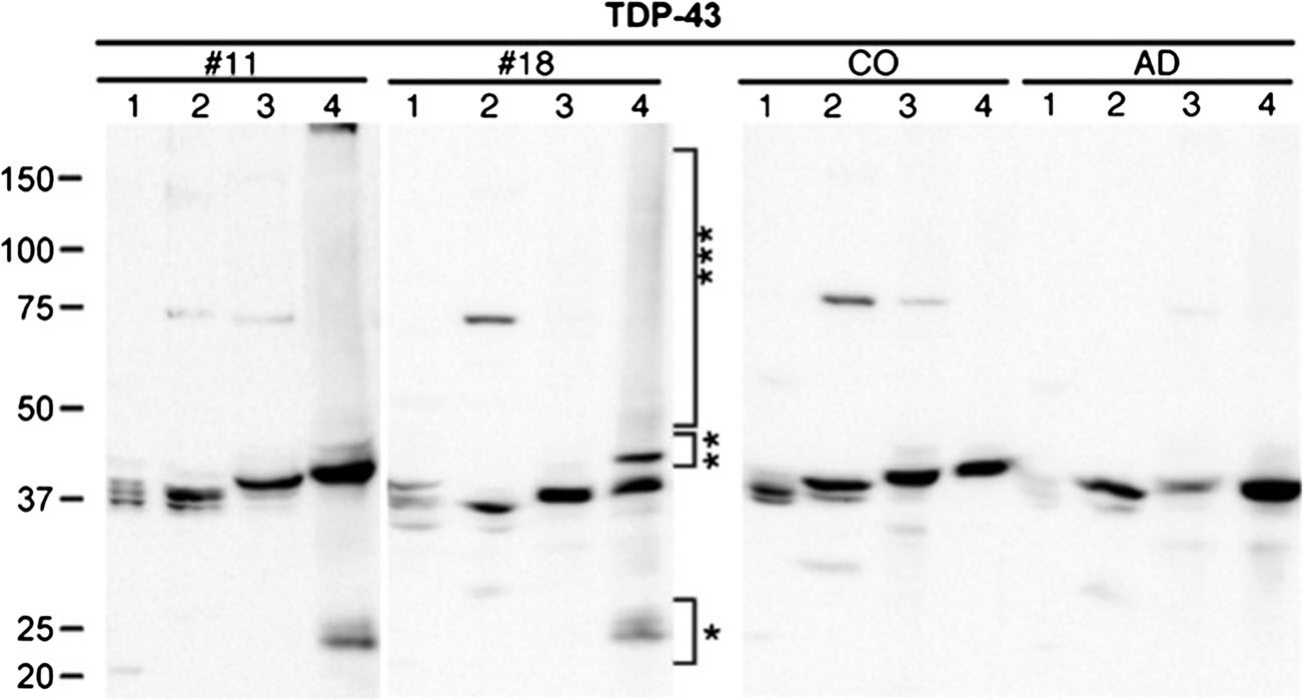
Immunoreactive pattern of TDP-43 in extracts from human brain tissue of sporadic and familial FTLD-TDP types 1 and 2 with an antibody against full-length TDP-43. From Neumann et al., Science, 2006 [6]. Reprinted with permission from AAAS. Immunoblots show pathologic 25-kD bands (*), 45-kD bands (**), and high molecular smears (***) in the urea fraction. *Lane 1* low salt, *lane 2* high salt with Triton X-100, *lane 3* sarkosyl, *lane 4* urea

In nearly all ALS and Tau-negative FTLD cases, TDP-43 can be found pathologically aggregated in the cytoplasm of neurons and glial cells. Immunoblotting of sarkosyl-insoluble urea-soluble fractions extracted from FTLD-TDP brains define a disease-specific signature for TDP-43 including a high molecular weight smear, phosphorylated full-length TDP-43 with a molecular mass size of 45–50 and 60 kDa, and truncated forms at 24–26 kDa, identified as C-terminal fragments of TDP-43 (Fig. 3) [6, 47]. Importantly, both the higher molecular weight bands and the lower truncated forms are phosphatase sensitive, implying disease-associated hyperphosphorylation. Phosphorylation sites of TDP-43 are mostly located at serine and threonine residues of the C-terminal glycine-rich domain of the protein (Fig. 1) [8, 48]. Ubiquitin-positive inclusions stain strongly for TDP-43 phosphorylated at serine residues 379, 403/404, 409, 410, and 409/410 [9]. However, it remains to be determined whether phosphorylation of TDP-43 is an early event in disease pathology, perhaps even promoting mislocalization and aggregation, or is secondary to aggregate formation and degradation processes [49–51].

A post-translational modification affecting only the full-length form of TDP-43 is the acetylation of lysine residue 145 of the RNA-binding motif [10]. Through a disruption of its physiological function in binding RNAs, unbound TDP-43 becomes more vulnerable to aggregate formation under prolonged stress conditions [10].

The phosphorylated form of TDP-43 can be found distributed in a relatively distinct anatomical pattern in ALS postmortem brain tissue, which has led to the definition of four neuropathological stages [52]. In stage 1, phosphorylated TDP-43 is mostly present in the spinal cord and the motor cortex with later stages involving frontal and temporal lobe structures, with widespread cerebral pathology including the neocortex and allocortex in stage 4. A similar regional pattern has been described for the behavioral variant of FTD, where stage 1 describes the lowest pathological burden, stage 2 additional frontal and temporal lobe involvement, while cortex and spinal cord TDP-43 pathology is seen in later stages [53]. All stages describe neuropathological patterns at postmortem and have not yet been convincingly linked to clinical severity of disease or functional decline in vivo. Within the clinical spectrum of FTLD, different TDP-43 pathological subtypes are observed and categorized as FTLD-TDP types A–E. Often, a pathological subtype can be related to a certain clinical phenotype or gene mutation [54–56]. FTLD-TDP type A consists of neuronal cytoplasmic inclusions and dystrophic neurites mainly located in cortical layer II, and is often associated with behavioral variant of FTD and an underlying mutation in the progranulin gene. Seen in ALS and FTD-ALS, type B describes moderate neuronal cytoplasmic inclusions and dystrophic neurites which are present in all cortical layers, while skein-like inclusions are typically seen in the spinal cord.

In addition to the phosphorylated full-length TDP-43, truncated C-terminal fragments of TDP-43 can be found in cytoplasmic inclusions. In postmortem brains, the distribution of the truncated C-terminal fragments of TDP-43 has been found to mainly involve the cortex and hippocampus, in a similar pattern to phosphorylated full-length TDP-43. In contrast, spinal cord inclusions stain for both the N- and C-terminal ends of TDP-43, suggesting the predominant presence of the full-length form [57]. The presence of C-terminal fragments in sarkosyl-insoluble urea-soluble fractions of the spinal cord is less frequent and more variable compared with fractions of brain tissue [6]. The length of the C-terminal fragments may also differ between different types of TDP-43 pathology. While shorter fragments are observed in FTLD-TDP types A–C, longer C-terminal fragments may predominate in a recently described FTLD-TDP subtype E [55]. Acetylated TDP-43 is only found in the spinal cord of ALS patients and not in the brain, where C-terminal fragments predominate [10].

A detailed insight into pathological forms of TDP-43 from sarkosyl-insoluble brain fraction of two ALS cases was gained by proteomic analysis. A phosphorylation-dependent TDP-43 antibody strongly reacted with pathological TDP-43 bands (phosphorylated 45-kDa band) and additional C-terminal fragments at 18–24 kDa, which were not detected in control brains. By extraction of those phosphorylation-dependent bands, subsequent mass spectrometric analysis revealed several intrinsically cleaved peptides, N- and C-terminal peptides, and modifications of sarkosyl-insoluble TDP-43. Almost all modifications were localized in the Gly-rich C-terminal half. Importantly, several novel phosphorylation, deamidation, and cleavage sites were identified by this approach. Common modifications focused on the 180–330 residue region, suggesting that this region of TDP-43 is affected in disease [8].

Cell-specific differences in TDP-43 distribution and in the propensity to form inclusions within regions affected by early TDP-43 pathology have been reported. In α-motoneurons of the spinal cord, loss of normal nuclear TDP-43 is always accompanied by the formation of cytoplasmic TDP-43 deposits, either granular or compact which form phosphorylated skein-like TDP-43 inclusion bodies in advanced stages. While in large Betz cells of the motor cortex, only sparse cytoplasmic deposits can be found, which are not phosphorylated [58]. This finding might imply that cytoplasmic TDP-43 in affected Betz cells is still soluble, or in a state not detected by the available antibodies. Considering that both cells are connected by corticospinal projections, together with the simultaneous appearance of TDP-43 pathology in distant regions, axonal trafficking of TDP-43 may be an important pathological pathway [59]. This might support an anterograde model of pathological propagation (“top-down”), if Betz cells are somehow more able to solubilize the abnormal TDP and continue to function, while passing it on to distal, less tolerant LMNs.

## Are Disease-Associated TDP-43 Isoforms Reflected in Biofluids?

The distribution of the pathological isoforms of TDP-43 has been well established from human postmortem tissue. All TDP-43 antibodies applied in biofluid studies so far bind to aa1–260, 205–222, or 256–296 of TDP-43 [60] and therefore have the potential to detect full-length TDP-43, longer C-terminal fragments, or phosphorylated full-length TDP-43. None of these antibodies bind to the extreme C-terminus of TDP-43, which makes it unlikely to detect shorter C-terminal previously described from human brain tissues (see Fig. 1). Only antibodies which bind to the phosphorylated serine at aa409/410 of the C-terminus of TDP-43 are likely to detect both pathological forms of TDP-43. However, immunohistochemical staining revealed unspecific binding of phosphorylation-dependent antibodies to the physiological nuclear TDP-43 [61]. While in neuropathological studies this antibody seems to specifically stain cytoplasmic aggregated TDP-43, it is not clear if in the presence of all forms of TDP-43 the binding is specific for pathological TDP-43.

**Fig. 4:**
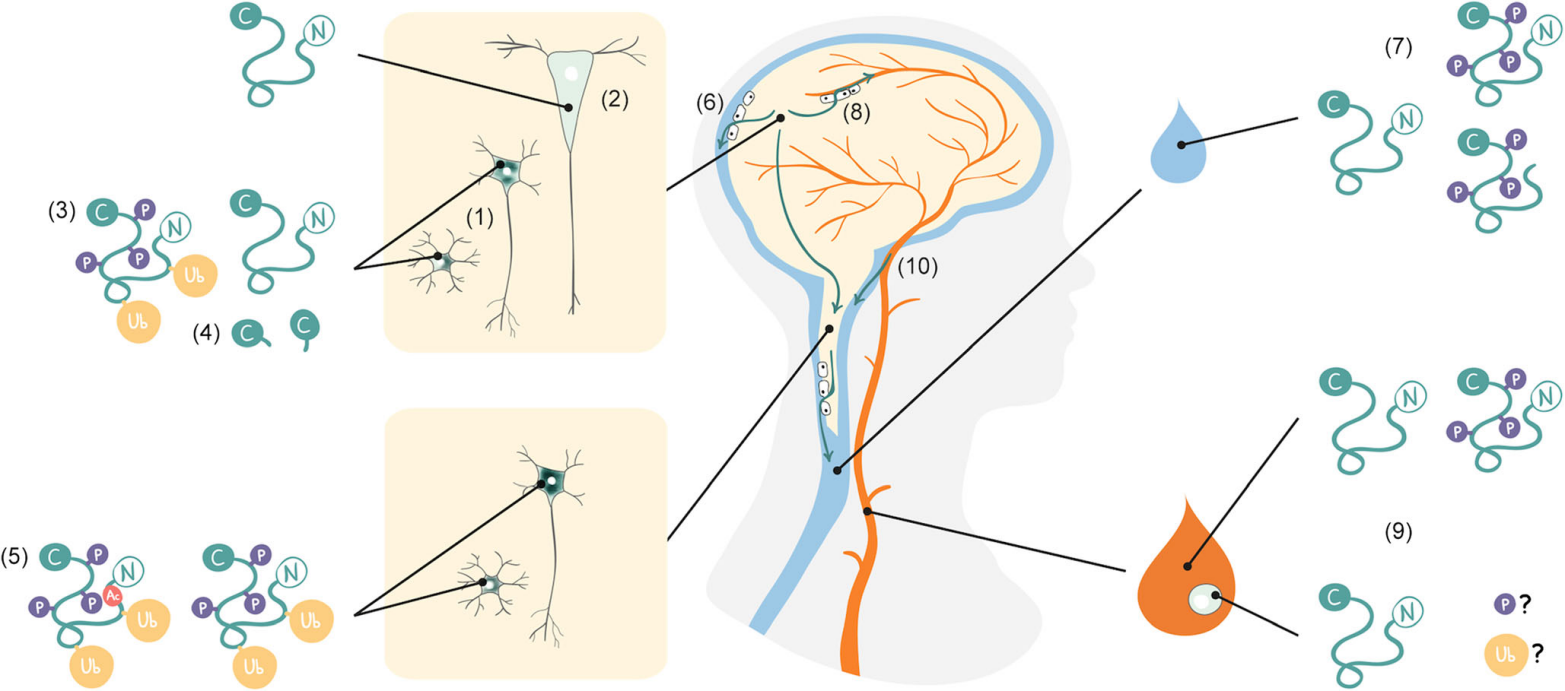
TDP-43 pathology and trafficking in amyotrophic lateral sclerosis and frontotemporal dementia. (1) Neuronal and glial aggregates of TDP-43 are formed by mislocalization of the normal nuclear protein to the cytoplasma. (2) In contrast to spinal motor neurons, in Betz cells, only sparse cytoplasmic deposits of TDP-43 can be found. (3) Aggregated TDP-43 is pathologically altered by phosphorylation and ubiquitination and (4) additional C-terminal fragments of TDP-43 are detected in the brain. (5) In addition to phosphorylation and ubiquitination, acetylated TDP-43 is found in the spinal cord of ALS. (6) The cerebrospinal fluid (CSF) compartment is in direct contact with the central nervous system and enables brain-derived proteins to transfer along the brain-CSF barrier. (7) Both normal full-length and phosphorylated TDP-43 are detected while the n-terminal end of TDP-43 has not yet been detected in CSF. (8) A tight epithelial barrier separates blood from brain. (9) A proportion of the full-length and phosphorylated TDP-43 can be measured in blood and lymphomonocytes, which (10) might also derive from CSF by diffusion along the blood-CSF barrier

Disease-specific biomarkers have been highly successful in other neurodegenerative diseases such as Alzheimer’s disease, where measurement of neuron-specific proteins such as protein tau and amyloid-ß have provided a high sensitivity and specificity of diagnostic tests [62]. However, TDP-43 is an ubiquitous expressed protein and the measurement of the brain-specific form of TDP-43 in complex biofluids such as cerebrospinal fluid (CSF) and blood is challenging. The most easy accessible biofluid is blood; however, protein exchange between the brain and blood is highly regulated through epithelial tight junctions and brain-derived proteins are expected to be much lower abundant, while the CSF compartment is in direct contact with the brain and therefore best reflects brain-derived proteins [63]. Nevertheless, depending on their hydrodynamic radius, proteins also diffuse along the blood-CSF barrier [64, 65] and blood-derived proteins may influence CSF TDP-43 homeostasis. An overview of TDP-43 pathology and suggested trafficking of TDP-43 is shown in Fig. 4.

### Cerebrospinal Fluid

The pathological signature of TDP-43 seen in immunoblots of postmortem tissue has been only partially reproduced in CSF from patients with ALS and FTLD [66]. A polyclonal antibody recognizing aa1–260 of TDP-43 detected a 45-kDa band (see Table 1). This molecular weight is slightly higher than the expected molecular weight of 43 kDa of native TDP-43 and therefore suggests the presence of posttranslational modified full-length TDP-43. However, so far, it has not been possible to detect an immunoreaction with a phosphorylation-dependent antibody for TDP-43 in CSF. The immunoreaction of TDP-43 at 45 kDa was also detected by an antibody against the C-terminal of TDP-43, but not by the N-terminal specific antibody. A possible explanation is N-terminal truncation of full-length TDP-43, as has been suggested by the authors, but the lack of a shift to a lower molecular weight may argue in favor of a recently described mechanism of physiological N-terminal mediated oligomerization with impaired antibody binding [67]. An additional band was detected by the C-terminal antibody at about 20 kDa; however, this band was also evident in healthy controls. Therefore, the pathological signature of C-terminal fragments of TDP-43 between 24 and 26 kDa has not been reproduced in CSF. The significance of the main finding that TDP-43 is detected in CSF at higher level in ALS/FTD is unclear, given that the same immunoreactivity for suggested phosphorylated full-length TDP-43 is also detected in disease controls [66].

**Table 1:** Qualitative immunoreaction of TDP-43 in biofluids

Study	Detection AB TDP-43	Patients	Number	Results
Steinacker (2008)	rab poly FL (aa1–260) 10782-2-AP, Proteintech, USA gl mouse mono (aa205–222) H00023435-M01, clone 2E2-D3, Abnova, Taiwan c-term (aa396–414) n-term (aa6–24)	FTD ALS ALS-DI ALS-FTD CON	12 15 3 9 13	Poly FL: 45 kDa and unspecific IgG 28 kDa c-term: 45 kDa and 20 kDa Mono FL and n-term: n.d.
De Marco (2010)	mouse mono (aa205–222) H00023435-M01	ALS TARDBP CON	17 4 13	Nuclear to cytoplasmatic immunoreactivity shift in ALS blood lymphomonocytes
Feneberg (2014)	rab poly FL (aa1–260) 10782–2-AP c-term (aa396–414)	ALS FTD CON	9 4 8	Poly FL: 45 kDa and unspecific to IgG and albumin 1/200 CSF/serum ratio

*FTLD* frontotemporal lobar degeneration, *FTD* frontotemporal dementia, *ALS* amyotrophic lateral sclerosis, *CON* controls, *DI* disinhibition, *poly/mono FL* polyclonal/monoclonal full-length TDP-43 antibody, *c-term* c-terminal antibody, *n-term* n-terminal antibody, *n.d.* not detectable

### Blood Lymphocytes

Given that it is a ubiquitously expressed protein, other studies have pursued the possibility of TDP-43 as a biomarker in blood lymphocytes [68]. Blood from ALS patients and a small subgroup of C-terminal TDP-43 mutation carriers was compared to asymptomatic first-degree relatives with and without mutations, and to healthy controls. Lymphocytes were isolated and cellular and nuclear fractions were prepared prior to Western blotting. Immunoreactivity of nuclear TDP-43 was shown close to the physiological molecular weight at 44 kDa, reflecting full-length TDP-43. In contrast to what is observed in subcellular brain fractions, cytoplasmic TDP-43 in lymphocytes had a slight shift to a lower molecular weight at 43 kDa. Interestingly, in ALS patients, and the case of an asymptomatic TDP-43 mutation carrier, there was a shift of TDP-43 to the cytoplasmic compartment (although the overall quantity of cytoplasmic TDP-43 was much lower than in the nuclear fraction). This finding suggests the presence of pathological mislocalization of TDP-43 from the nucleus to the cytoplasm and reflects nuclear clearance in ALS. This shift was not observed in a patient with a *SOD1* mutation confirming that this subgroup of ALS is associated with a different molecular pathway.

### Blood and CSF TDP-43 Isoform Comparison

The TDP-43 biochemical profile detected in both blood and CSF was investigated to see whether it reflected brain-derived TDP-43, or predominantly represents the physiological full-length form derived from blood [69]. The study demonstrated that similar 2-D immunoreactive patterns of TDP-43 at 45 kDa were present in CSF and serum, whereas in brain tissue of ALS patients with TDP-43 pathology, a higher and more acidic pattern was observed at 50 kDa, suggesting post-translational modifications in addition to the full-length form of TDP-43. The ratio between TDP-43 concentrations in CSF and serum seemed to be similar to proteins of the same molecular weight, which easily diffuse along the blood-CSF barrier, making it likely that the TDP-43 detected in CSF was merely blood derived. Exosomes as transporters of cellular proteins which are released into the extracellular compartment may in general be a promising substrate for the identification of those intracellular mislocalized proteins involved in neurodegenerative diseases [70]. Evidence using an in vitro over-expression model described the transmission of TDP-43 via exosomes obtained from primary cortical neurons that may contain the pathologically phosphorylated form of TDP-43 [71].

## Quantitative Measurement of TDP-43 in CSF and Blood

Given the indications of altered TDP-43 in biofluids, subsequent studies have attempted to quantify the degree of TDP-43 and the pathological altered TDP-43 using either a monoclonal or polyclonal TDP-43 antibody against amino acid regions 1–260, 205–222, and 256–296 or phosphorylation-dependent antibodies to aa409/410 of TDP-43. Nearly all studies used enzyme-linked immunosorbent assay (ELISA) to measure TDP-43 in CSF and plasma of different patient groups. An overview of all ELISA studies is given in Table 2.

**Table 2:** TDP-43 ELISAs in biofluids from 2008 to 2017

Study	Capture TDP-43 ab	Receptor TDP-43 ab	ELISA detection (limit)	Patients	Number	Results FL TDP-43	Results pTDP-43
Cerebrospinal fluid							
Kasai (2009)	Mouse mono (aa205–222) H00023435-M01, clone 2E2-D3, Abnova, Taiwan	rab poly FL (aa1–260) 10782–2-AP, Proteintech, USA	Chemiluminescence Light unit to concentration (ng/ml) (> 0.49 ng/ml)	ALS CON	30 29	6.92 ± 3.71 ++ 5.31 ± 0.94	
Noto (2011)	Mouse mono (aa205–222) H00023435-M01	rab poly FL (aa1–260) 10782–2-AP	Chemiluminescence Light unit to concentration (ng/ml)	ALS PD, MSA, PSP GBS MS	27 15 20 15	29.5 ± 15.5 ++ 19.7 ± 6.6 16.7 ± 7.5 13.7 ± 9.0	
Junttila (2016)	n/a Kit from Cusabio, China	Biotinylated n/a	Streptavidin-conjugated HRP Optical density to concentration (> 0.078 ng/ml)	FTD FTD C9orf72 ALS ALS C9orf72	49 20 11 10	3.0 ± 1.1 2.9 ± 0.7 3.7 ± 1.0 4.6 ± 1.5 ++	
Kuiperij (2017)	*Ventricular* Goat poly (aa256–269) Abcam, UK/ Rat poly pTDP (S409/S410) Merck Millipore, USA	rab poly FL (aa1–260) 1782–2-AP/ Biotinylated rab poly FL	Chemiluminescence Light unit to concentration (ng/ml) Streptavidin-conjugated HRP	FTLD-TDP FTLD-Tau AD AD-TDP CON	15 14 12 6 19	0.7 ± 0.3 0.9 ± 0.4 0.8 ± 0.6 0.6 ± 0.5 1.5 ± 0.9 ++	1.5 ± 1 1.6 ± 0.7 1.4 ± 1 1.5 ± 1.6 3.7 ± 3.5 ++
	*Lumbar* Goat poly FL/ Rat poly pTDP	Biotinylated rab poly FL	Streptavidin-conjugated HRP	FTLD-TDP FTLD-Tau CON	36 7 21	421 ± 335 407 ± 196 541 ± 108 ++	n.d. n.d n.d.
Plasma							
Foulds (2008)	Mouse mono (aa205–222) H00023435-M01	rab poly FL (aa1–260) 10782–2-AP	Optical density (OD) (> 0.11 OD)	FTD AD CON	35 102 85	0.26 ± 0.52 ++ 0.13 ± 0.33 0.02 ± 0.32	
Foulds (2009)	Mouse mono (aa205–222) H00023435-M01	Rab poly FL (aa1–260) 10782–2-AP/ Mouse poly pTDP (S409/410–2) Cosmobio Co Ltd., Tokyo	OD	FTLD-TDP FTLD-Tau AD AD-TDP	16 12 16 8	0.52 ± 0.35 ++ 0.34 ± 0.18 0.40 ± 0.35 0.43 ± 0.34	1.31 ± 0.63 ++ 1.07 ± 0.50 1.05 ± 0.75 1.03 ± 0.64
Verstraete (2012)	Mouse mono (aa205–222) H00023435-M01	Rab poly FL (aa1–260) 10782–2-AP	Chemiluminescence Light unit to concentration (μg/l) (> 0.31 μg/l)	ALS CON	219 100	10.85 ++ 3.76	
Suarez-Calvet (2014)	Rab poly FL (aa1–261) 10782–2-AP (TDP-43 KE00005 Kit)/ rab poly FL (E9442h EIAab Kit, Wuhan)	Biotinylated mouse mono (aa203–212) 60019–2-Ig, Proteintech/ ab poly pTDP (S409) SAB4200223, Sigma-Aldrich, USA	Streptavidin-conjugated HRP Light unit to concentration Normalized to control sample	FTD FTD C9orf72 FTD GRN CON	51 10 5 22	5.9 (3.6–9.4) ++ 1.5 (1.0–7.2) 0.8 (0.5–5.3) 4.4 (3.2–6.9)	0.12 (0.02–0.8) 1.6 (0.3–9.7) 2.9 (1.0–8.7) ++ 0.05 (0.0–0.3)

*FTLD-TDP* Tau autopsy-confirmed frontotemporal lobar degeneration with TDP-43/Tau pathology, *FTD* frontotemporal dementia, *ALS* amyotrophic lateral sclerosis, *AD* Alzheimer’s disease, *AD-TDP* with TDP-43 pathology, *CON* controls, *GRN* progranulin gene mutation, *poly/mono FL* polyclonal/monoclonal full-length TDP-43 antibody, *pTDP* phosphorylated TDP-43 antibody. Results are given as mean ± SD and confidence intervals (). *n.d.* not detectable. ++ highest value. *n/a* not available

### Quantification of TDP-43 in CSF

The first quantification of TDP-43 in the CSF of ALS patients used an ELISA technique against full-length TDP-43 with a commercially available chemiluminescent substrate to enhance the TDP-43 signal [72]. With a detection limit of 0.49 ng/ml, there was a significant difference between apparently sporadic ALS patients and a group of neurological and healthy control subjects. Six ALS patients with a disease onset within 10 months of sample collection had high levels over the 95% CI of the control group, while all other samples were within the range of TDP-43 levels seen in the controls, suggesting higher levels in the early phase of disease. The elevation of TDP-43 did not correlate with the level of diagnostic certainty (using the El Escorial criteria for ALS) nor with the site of disease onset (limb vs bulbar). The same methodology was used to measure TDP-43 levels in the CSF of ALS patients compared to other neurodegenerative diseases such as Parkinson’s, and also neuroinflammatory disease [73]. Mean TDP-43 levels were higher in the ALS group than in all the control groups. A cutoff level of 27.9 ng/ml with a sensitivity of 59.3% and a specificity of 96.0% was suggested for ALS. Despite the overall picture of higher TDP-43 in other studies, lower TDP-43 levels were found to correlate with a shorter survival. A commercially available ELISA with a biotin-streptavidin method for the detection of TDP-43 has since been used to distinguish a group of FTD patients from a smaller group of ALS patients and *C9orf72* repeat expansion carriers of both subgroups [74]. TDP-43 levels were higher in ALS patients, regardless of genotype or clinical features; however, the ALS group was smaller (*n* = 10) relative to the FTD patients (*n* = 50). In contrast to previous studies, a positive correlation with male gender was observed. However, it should also be noted that the study also reported a high inter-assay coefficient of variation.

Another study group used an ELISA with a full-length TDP-43 coating antibody for ventricular CSF obtained postmortem at the time of autopsy and lumbar CSF samples, applying a biotin-streptavidin detection method for the latter [75]. For each ventricular CSF sample, neuropathology was confirmed as FTLD-TDP, FTLD-Tau, or AD, with and without TDP-43 pathology at autopsy. The lumbar CSF was obtained from a patient group determined as FTLD-TDP or AD either by autopsy, by genetics (*GRN*, *C9orf72*, and *MAPT*) or clinically. Remarkably, this is the first study reporting *lower* TDP-43 levels in patients with neurodegenerative diseases of FTLD-TDP and AD compared to controls. Thus, in lumbar CSF, full-length TDP-43 was significantly lower in FTLD-TDP compared to controls, but with high group variability, while FTLD-Tau was not different from controls or FTLD-TDP. In lumbar CSF, phosphorylated TDP-43 was not detectable. In autopsy-obtained ventricular CSF samples, lower levels of full-length TDP-43 were detected in FTLD-TDP and AD compared to controls, and non-significantly lower levels in FTLD-Tau were observed. Phosphorylated TDP-43 levels were lower in FTLD-Tau and AD, and a trend towards lower levels was observed in FTLD-TDP. This study might suggest that lower levels of TDP-43 are a feature of neurodegeneration in general, as lower levels were not restricted to TDP-43 proteinopathies. Of note, a possible breakdown of the CSF barrier postmortem is likely to have an influence on the quantification of TDP-43 in the ventricular CSF samples such as impaired antibody binding through higher IgG and albumin contamination from blood [75].

### Quantification of TDP-43 in Plasma

The first study to quantify the full-length TDP-43 isoform in plasma included an initial patient cohort clinically diagnosed with FTD and Alzheimer’s disease (AD) [76]. In this study, 22% of AD patients and 46% of FTD patients were above the detection limit of the assay, with most healthy control samples (92%) falling below this level. A subsequent study by the same group included plasma samples from autopsy-confirmed cases, and quantified both full-length and phosphorylated TDP-43 as the detection antibody [77]. Full-length TDP-43 concentrations did not differ between FTD and AD patients, including when patients documented to have TDP-43 pathology were considered as an independent group. Phosphorylated TDP-43 levels were non-significantly higher in FTD patients with TDP-43 pathology than FTD with Tau pathology, but not in clinical AD patients with TDP-43 pathology. Phosphorylated plasma TDP-43 levels in FTD cases were positively correlated with the severity of TDP-43 pathology, but not in clinical AD with TDP-43 pathology. Overall, a wide variation in individual levels was observed.

In a separate study, plasma levels of TDP-43 in a large cohort of 219 ALS patients were measured by ELISA using a chemiluminescent substrate [78]. By this method, 28% of ALS patients and 21% of control subjects were over the detection limit. Overall, TDP-43 levels in ALS, including three patients with *TARDBP* mutations, were increased compared to healthy control subjects. A positive correlation with age was shown. A subgroup of patients had longitudinal samples at different time points but no conclusion could be drawn due to the heterogeneity of TDP-43 levels.

Another study measured full-length and phosphorylated TDP-43 in plasma in FTD non-mutation carriers compared to a small subgroup of FTD patients with a *C9orf72* repeat expansion, *GRN* mutation carriers, and healthy controls [79]. This study applied a commercially available ELISA with a biotinylated detection antibody against the phosphorylated TDP-43 and streptavidin-conjugated chemiluminescence. Concentrations were normalized to a control sample and given as relative units compared to this internal standard on each plate. Higher levels of phosphorylated TDP-43 were found in FTD *C9orf72* and *GRN* mutation carriers compared to FTD non-mutation carriers and healthy controls. Interestingly, full-length TDP-43 levels were found to be negatively correlated with phosphorylated TDP-43, meaning that it was lower in mutation carriers compared to non-mutation carriers and controls. Both TDP-43 forms in plasma correlated negatively with age, which was taken into account in the statistical analysis. No difference was found when investigating a smaller CSF sample group of 20 FTD patients and 5 FTD mutation carriers compared to controls.

A different methodology aimed to detect TDP-43 with an atomic force microscopy-based biopanning protocol, utilizing phage-displayed, single-chain antibody fragments directed against TDP-43 from an established library [80, 81]. Phage scFvs were biopanned against immunoprecipitated TDP-43 from homogenate samples of the motor cortex from healthy controls, ALS, and FTLD-TDP brains. The binding capacity of certain scFvs was shown to be specific only for ALS or FTLD-TDP brains, raising the possibility of different TDP-43 isoforms underlying both TDP-43 pathologies.

## Limitations of the Quantification of TDP-43 in Human Biofluids

The pathologic specimens of TDP-43 have been well established from human postmortem studies; however, the quantification of TDP-43 isoforms in the CSF and plasma of ALS/FTLD patients has been contradicting. Although a range of antibodies can be used to detect TDP-43 in biofluids [82], an unacceptably high level of variability of detection in samples from ALS and FTLD patients has been reported. Quantification of TDP-43 and even more its phosphorylated form in CSF appears limited by very low concentrations or low binding affinity of the antibodies in the presence of high abundant immunoglobulines and albumin. While most of the earlier studies detect higher TDP-43 levels in CSF and plasma for the suggested full-length form of TDP-43, later studies report lower TDP-43 plasma levels in FTD patients that were more likely to have underlying TDP-43 pathology compared to a more randomly selected FTD patient cohort, and controls [79]. Lower TDP-43 levels in CSF have also been observed in mutation carriers with underlying TDP-43 pathology and in ALS patients with a shorter survival [73, 75]. Both studies however were relatively small and heterogeneous, and more systematic investigation is required. In summary, it is not yet clear if TDP-43 rises due to degeneration of nerve cells and release of intracellular proteins or if it is reduced due to aggregate formation.

A limitation of all reviewed ELISA studies on plasma and CSF TDP-43 levels is the use of the common commercially available antibodies for TDP-43. These are restricted to a certain peptide region or phosphorylation sites of TDP-43. Immunoblotting shows that the identification of TDP-43 in CSF and serum by the commonly applied antibody combinations used for quantification represent a 45-kDa full-length form of TDP-43, rather than disease-specific truncated forms of TDP-43 while even more concerning commonly applied phosphorylation-dependent antibodies have not yet revealed a convincing immunoreaction in blood or CSF [66, 69]. Proteomic identification by mass spectrometry of TDP-43 peptides present in biofluids from 1D or 2D immunoblotting gels or in-solution is still outstanding and may be due to insufficient affinity purification of TDP-43 from complex biofluids prior to mass spectrometry which highly relies on specific antibody binding to TDP-43.

Monoclonal antibodies which selectively recognize the pathological forms of TDP-43 will be of critical importance for the monitoring of disease status and to assess future treatment options (such as immunization studies). An extensive study generated new monoclonal antibodies by immunization of recombinant full-length, N-terminal, and C-terminal TDP-43 peptides recognizing epitopes distributed over the entire length of TDP-43. Although many of these antibodies are suitable for common applications (ELISA, WB, IHC), there was only one antibody reported “mab138” against aa182–261 (including the RNA binding motif 2 and the nuclear localization signal), which appeared to detect cytoplasmic, pathological TDP-43, independent of its phosphorylation status and not endogenous TDP-43 [61].

### Future Directions

To apply disease-specific TDP-43 antibodies in future studies will be essential to detect brain-derived TDP-43 and to limit the detection of normal TDP-43, which may derive from blood [69]. These studies may inform about different TDP-43 types among specific ALS/FTLD genotypes and across different TDP-43 pathology subtypes. Rather than using disease models of TDP-43 such as transient over-expression or recombinant proteins for antibody development, recent data suggests that it might be necessary to specifically isolate the pathological forms of TDP-43 from ALS and FTD postmortem tissue and biofluids. To unravel the biochemical molecular pathology of TDP-43, it might be even necessary to investigate TDP-43 on a single cell or brain-specific level in diseased tissue [83]. This may lead to the identification of new or early disease-specific changes, potentially missed in whole tissue analysis or cell culture over-expression models. Mass spectrometry analysis is a powerful method used to identify proteins or peptide sequences and characterize modifications without the limitations of antibody-based methods [84, 85]. However, to target a specific protein for peptide identifications, the complexity of biofluids requires the enrichment of the target protein prior to mass spectrometry analysis. Pathological modifications identified from *postmortem* tissue such as phosphorylation and ubiquitination are potential candidates to enrich pathological TDP-43 forms. Serial fractionation of postmortem tissue enables to isolate and enrich insoluble TDP-43 with the identification of a disease-specific pattern [6, 8, 47]. Therefore, free and soluble TDP-43 in biofluids may not represent the pathological form of TDP-43 [69, 82], and the release of aggregation-prone cytoplasmic TDP-43 from affected motor neurons may even result in insoluble TDP-43 fractions requiring extraction [86]. Only through the identification of pathological TDP-43 peptides the development of quantitative assays will be informative in future. Assay development on the new digital analytical platforms, e.g., Simoa or Mesoscale, will then provide a useful tool for a more sensitive detection of low abundant pathological forms [87–89]. As so far the production of protein-based antibodies is cost and time intensive and the binding capacity of protein-based antibodies against TDP-43 may also be influenced by protein polymerization leading to epitope masking of the detection region, aptamers which are small DNA or RNA molecules are new antibodies selected through a faster in vitro screening process known as systematic evolution of ligands by exponential enrichment (SELEX) and able to recognize specific amino acid sequences of proteins. Aptamers can be chemically modified (e.g., with fluorescent dyes or compounds) for the development of in vitro labeling of TDP-43 or in vivo drug delivery [90, 91]. Their binding affinity is high and conformation dependent, which may be of interest when studying oligomerization of proteins [92]. Recombinant TDP-43 forms oligomers and fibrils in vitro; however, the mechanisms and the involved domains are still unknown and the detection of TDP-43 oligomers so far is restricted to FTLD-TDP brain tissue [45]. The change of the conformational state of TDP-43 by mutations in its primary structure or the introduction of modifications and the formation of ß- and amyloid-like structures, together with evidence for oligomerization and cell-to-cell transmission, implies a disease-specific readout known from other neurodegenerative diseases [22, 45, 67, 93, 94]. Biomarker discovery may also be advanced through the study of proteins which interact with the pathological forms of TDP-43 [95, 96]. New cell models (human-induced pluripotent stem cells) and genetic engineering to maintain the biologic expression ratio of the protein of interest have the potential to verify the most relevant interactors, which can then be translated to patient biofluid investigations [97]. TDP-43 interacts directly with proteins involved in nuclear splicing of RNA or in cytoplasmic processing of mRNA, for example in stress granules. These functions might be directly affected if either mutations or cytoplasmic aggregation alter the TDP-43 protein interactome. Therefore, the identification of distinct interactions of pathological TDP-43 or downstream effects on RNA may reveal other proteins or RNAs with value as markers of disease activity [16, 95].

TDP-43 is central to ALS and FTLD pathology, and the frontrunner for a suitably specific biomarker that may also reflect individual disease activity. While the simple measurement of TDP-43 from blood and CSF has failed so far, only continued advances in understanding the localization, protein interactions, and immunology of its various modified forms will deliver this full potential.
